# Determinants of efficiency growth of county-level public hospitals-evidence from Chongqing, China

**DOI:** 10.1186/s12913-019-4609-9

**Published:** 2019-11-21

**Authors:** Jing Liu, Beibei He, Xiaolan Xu, Leming Zhou, Jiang Li, Gongru Wang, Yingyao Chen

**Affiliations:** 1 Key Lab of Health Technology Assessment, National Health Commission, School of Public Health, Fudan University, Shanghai, 200032 People’s Republic of China; 2 Department of Health Management, School of Management, Hainan Medical University, Hainan, 571199 People’s Republic of China; 3 Hospital Management Institute, Fudan University, Shanghai, 200032 People’s Republic of China; 4 Department of Statistics and Development Research, Chongqing Health Information Center, Chongqing, 401120 People’s Republic of China

**Keywords:** County hospital efficiency, Three-level growth model, Longitudinal data, Chongqing

## Abstract

**Background:**

The reform of county-level public hospitals is a breakthrough in the new era of healthcare reform in China and has attracted considerable attention since 2012. Continuous and efficient operations of hospital are primary concerns of this reform. To ensure the effectiveness of county-based intervention reform measures in Chongqing, it is significant to understand how hospital and county characteristics are associated with county-level public hospital efficiency due to significant development differences between counties. This study identifies the trajectory of hospital efficiency over time and determinants. It will also provide preliminary references for advancing reform.

**Methods:**

This study employs data from the Chongqing Regional Health Information Platform, Chongqing Health and Family Planning Statistical Yearbook, and Chongqing Statistical Yearbook for 2012–2016. A three-level growth model is used to estimate the efficiency growth trajectories within the contexts of hospitals and counties.

**Results:**

The intra-hospital level factors that affect the initial efficiency include government financial assistance, daily charge per bed, total assets turnover, number of hospital healthcare technicians, and medical costs per 100-yuan medical income. Inter-hospital variance is explained by hospital type. Inter-county level factors affecting the growth rate include the number of healthcare technicians per 1000 people and population density of the county. The interaction effect of the number of hospital healthcare technicians, hospital type, and number of healthcare technicians per 1000 people on hospital efficiency growth is significant.

**Conclusions:**

This study identifies determinants that contribute to efficiency changes in public county-hospitals over time by using a three-level growth model. The differences in efficiency are associated with intra-hospital, inter-hospital, and inter-county characteristics in Chongqing, which provides useful insight into government decision-making and the progress of reform. The stability and reasonable increase in the number of healthcare technicians in a county are the key factors that improve the efficiency. Further reform should focus on maternal and child healthcare hospitals for increasing investment and implementing government compensation.

## Background

Hospitals are one of the main components of the health system and also an important driver of increased health care costs constituting the largest single component of health expenditure in many countries [1]. They now face the daunting challenge of providing safe, effective care in complex organizations strapped by heavy patient loads, limited staffing, and shrinking financial resources [2]. Although the ratio of health expenditures to gross domestic product (GDP) in China has increased from 3.15% in 1980 to 6.36% in 2017 [3], the government is still confronted with an endemic public deficit. Under these circumstances, the new comprehensive nationwide health care reforms have been initiated by the Chinese government since 2009 and have focused on efficiency, quality, patient-centered care, and payment reforms [1]. In June 2012, the State Council issued the “Opinions on the Comprehensive Reform of Public Hospitals at the County Level” and determined that “the reform of public hospitals should take county hospitals as the breakthrough point,” thus directing the focus of the medical reform to county-level public hospitals.

County-level public hospitals play an important role in the urban and rural medical and health service system in China. There are three types in China—general hospitals (GHs), traditional Chinese medicine hospitals (TCMHs), and maternal and child healthcare hospitals (MCHHs). These serve more than 900 million people, accounting for more than 70% of county residents [4]. In comparison with community health service agencies, township hospitals, and other primary health institutions, county-level hospitals’ internal operation management, external policy environment, commitment to medical tasks, and structure of personnel resources are more complex and dynamic [5]. They have long confronted the challenges of poor medical equipment, outdated technology, and weak scientific research. The reform of county-level public hospitals is targeted at the supply-side to form a more scientific and regulated management system, and mechanisms for governance, compensation, and monitoring, and to improve internal management to upgrade operating performance for safe, high quality, cost-effective, efficient, and better services [1]. Optimizing county health resource allocation and continuous and efficient operations are important considerations of the reform [6].

Therefore, it is significant to understand the relative efficiency in healthcare resource utilization for the county so that the National Health Commission of the People’s Republic of China (NHCPRC) can develop targeted policy decision-making. Efficiency mainly examines the relationship between input and output. It is used to measure the extent to which resources are used effectively under given input conditions of an organization. Pareto efficiency is optimal under fully competitive market conditions and makes the most of the available resources. However, for public hospitals, in many cases, that do not have total competition in the marketplace, alternative strategies must be devised for improving efficiency in resource use [7]. Moreover, county-level public hospitals seeking to improve efficiency and maximize health outcomes must first address the question of how to identify determinants [8].

This study identifies determinants of the efficiency of county-level hospitals in Chongqing, China. Chongqing is located in the western part of China and has a population of 30 million. It is spread over a vast area, of which mountains account for 76% [9]. There are significant differences in the development of counties. In 2007, the State Development and Reform Commission officially established Chongqing as a pilot area for the overall development and reform of counties, along with Chengdu. Chongqing is required to comprehensively promote the reform of various aspects, in accordance with the requirements of the reform experiment, for the comprehensive planning of counties. In recent years, Chongqing’s government has taken various effective measures to promote the balanced development of counties and gradually narrow the gap, with the integration of county health as the focus. This requires equity of basic public health and the homogenization of basic medical services. However, due to significant differences between counties, the balanced development of county health services still faces great challenges. To continue developing reform measures that reduce the gap in county health services, it is important to understand whether there is a difference in the efficiency between county-level hospitals in different hospitals and counties, and what determinants influence them. Therefore, we propose the following research questions: How efficiency are county public hospitals? Has the efficiency improved since the new healthcare reform was initiated in 2012? In what ways have efficiency growth trajectories changed over time? Do these trajectories vary significantly across hospitals and counties? If so, are there any hospital or county characteristics associated with this variation? The study assumptions are as follows:hypothesis 1: there is a difference in hospital efficiency between hospitals and counties; hypothesis 2: county characteristics have an impact on efficiency; hypothesis 3: hospital characteristics have an impact on efficiency; hypothesis 4: intra-hospital characteristics have an impact on efficiency; hypothesis 5: intra-hospital, hospital, and county characteristics will affect the efficiency growth and can adjust the relationship between time and it. To answer the above questions and verify hypotheses, the present study examined the relationships among efficiency, intra-hospital, inter-hospital, and inter-county characteristics.

## Literature review

Hospital efficiency analysis is an important issue within the field of health economics, and while there is abundant literature on this subject [6, 10–30], only a handful of studies focus on identifying the determinants of hospital efficiency [16–22]. Moreover, there is little research on which hospital and county characteristics are associated with efficiency changes. A review of the literature on hospital efficiency identifies two contemporary approaches to measure hospital efficiency: parametric and non-parametric approaches [31]. Efficiency measurement methods have been continuously innovated, such as stochastic frontier analysis, data envelopment analysis (DEA), and the bootstrap method. The DEA method has received increasing attention and is a valuable efficiency measurement and benchmarking tool for most organizations, especially in the healthcare sector [8]. This methodology not only expands the applications of efficiency evaluation, but also increases the accuracy of efficiency measurements and provides a new perspective for the study of hospital efficiency. Simultaneously, the research objective has evolved from purely the measurement of efficiency to the discussion of factors affecting efficiency to provide a direction for efficiency improvement. Although some studies use ordinary least squares, Tobit analysis has been the most popular analytical method, wherein the output-based efficiency score or reciprocal of the input-based efficiency score is regressed on variables posited to affect efficiency [32, 33].

The relationships among hospital efficiency and hospital and regional characteristics are often analyzed separately [24, 27, 34]. Prior literature suggests that hospital size and average length of stay are negatively associated with efficiency, whereas occupancy rate, bed-to-nurse ratio, and nurse-to-physician ratio are positively associated with efficiency [6]. Small and rural hospitals are slightly less efficient compared with large and urban hospitals, and teaching hospitals are substantially more efficient compared with non-teaching hospitals [22]. Although such research has attracted increasing attention from scholars, there remains a lack of formal academic studies examining the efficiency of healthcare delivery systems across states or counties. Hussey et al. [35] note that, between 1990 and 2008, only four studies examined the geographical differences in healthcare efficiency. To date, no study has been conducted on county-level public hospitals’ efficiency change over time to identify variables that accurately predict change across states or counties. Moreover, analysis has been limited to cross-sectional models. Gearhart [36] hypothesized that cross-country healthcare efficiency rankings should not be the primary tool to drive reform and policy. For policy prescriptions based on efficiency rankings, one should look within a country by considering efficiency rankings among individual states or localities [36], because there is an objective correlation between individual states and localities. Ignoring the rankings will inevitably bias the analysis results. This highlights the potential for methodological improvement [37].

A multilevel growth model is an effective statistical model to solve the above contradiction with longitudinal data [38–40]. Over the past several decades, longitudinal designs for studying individuals’ growth and change have slowly become popular in the area of psychological well-being (e.g., [41, 42]), although they are rarely used in studies on hospital efficiency. To ensure the effectiveness of county-based intervention reform measures in Chongqing, it is important to understand how hospital and county characteristics were associated with county-level public hospital efficiency. Three-level growth model was used to describe and demonstrate the importance of examining both hospital and county characteristics related to efficiency over time. Through the analysis of different hospital and county characteristics, the effects of these characteristics on the initial efficiency and efficiency growth rate are revealed, and the interaction effects are tested. The variability in efficiency trajectories between hospitals and counties was a unique perspective. The characteristics of counties and hospitals were examined to determine if they explain variability across the average growth trajectories of counties and hospitals.

This study contributes to the current knowledge base by filling the abovementioned gap in literature using the Chongqing case, and presents an innovation by introducing the multilevel growth model into the area of hospital efficiency studies. It will also provide important references for policymakers and hospital managers.

## Methods

### Materials and methods

**Fig. 1:**
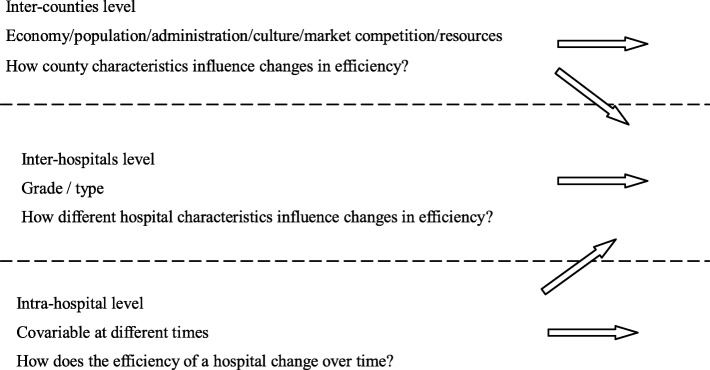
Defining variables in a three-level growth model [43]

To study the trajectory of hospital efficiency growth and the effects of hospital and county characteristics on efficiency, a three-level growth model was used. All analyses were conducted using the Mplus 8.0 software and the maximum likelihood robust estimator. In a three-level growth model (see Fig. 1),^1^ Level-1(Intra-hospital level) is a repeated observation at different times in the same hospital, observing the growth trajectory of each hospital over time; Level-2 (Inter-hospital level) is an inter-hospital observation that determines the heterogeneity of individual changes and how different hospital characteristics influence changes in efficiency; Level 3 (Inter-counties level) is a group variable that determines the heterogeneity of changes between counties [44].

A three-level growth model does not need to assume individual independence and can correct the bias of parameter standard error estimation caused by the non-independence of observation data. The effects of less- and highly independent variables on outcomes can be analyzed simultaneously. Random slope and cross-horizontal interactions can also be analyzed. The initial mean levels of the dependent variable and each explanatory variable can be obtained with parameter estimation results of the fixed component.

After considering the effect of different interpretation variables on the development differences between hospitals, three-level growth model use the difference between the interception and slope to explain the degree of change in the interpretation of the differences through parameter estimation of the random component [45]. Compared with traditional statistical methods, it is more flexible and has advantages in dealing with repeated measurement data.

### Sample and data sources

There are 38 counties in Chongqing totally. We implemented a purposive sampling design to obtain a representative sample of county hospitals throughout Chongqing, and to focus on 24 counties on the premise of balancing the geographical, economic, and service population characteristics, as well as hospital operations, according to the recommendation by the Chongqing Health Commission. The sample size was determined by data availability and the DEA method requires a complete set of input and output variables for study.^2^ Efficiency, Intra-hospital changes, hospital characteristics, and county characteristics were measured at five points from 2012 to 2016, forming a repeated measurement of annual data for five years. Relevant operational data were taken from the Chongqing Regional Health Information Platform. Information on county characteristics was supplemented from the Chongqing Health and Family Planning Statistical Yearbook and Chongqing Statistical Yearbook (2012–2016). Finally, 360 observations were obtained, and we had a multilevel longitudinal data set. No patient information was included in the study, so an ethics statement was not needed. An obvious feature of the above data was the multilevel nesting structure: observation time variables were nested in hospitals, and hospitals were nested in counties. The results in a sample of 72 hospitals were nested within 24 counties.

### Variables construction

Efficiency requires a production process [or unit] to maximize output for a given level of inputs [22]. Under the limited health resources in counties, the total market competition makes the resource utilization optimal and the efficiency increases gradually. An ability to operate efficiently often depends on the operational conditions and practices, such as the external operational environment in which production occurs, internal characteristics of firms, such as the type and vintage of technology, and managerial practices [46]. Therefore, the above measures of such contextual factors provide a better understanding of efficiency differences and identify the key efficiency determinants across hospitals. The factors of the geopolitical setting, economy, population structure and state of health, health resources, medical market competition, and demand for health services, as discussed in previous literature, pose challenges to healthcare delivery and access. Therefore, we hypothesize that these factors influence the efficiency of hospitals, and, thus, are included in our analysis.

The level-1 variables are measured over five time periods to determine how the efficiency of a hospital changed over five years. Following the literature review [47–49], level-1 variables include: financial assistance, medical business area, actual number of beds available, number of hospital healthcare technicians, total fixed assets, daily charge per bed, medical costs per 100-yuan medical income,^3^ total assets turnover, ratio of revenue and expenditure, and asset-liability ratio, on behalf of the hospital’s internal operation management. Level-2 variables identified how different characteristics of hospitals influence changes in hospital efficiency. These variables include hospital grade (unrated, middle second-class, upper second-class, middle first-class, and upper first-class hospitals) and type (GHs, TCMHs, and MCHHs). Level-3 variables identified how county characteristics influence changes in hospital efficiency. These variables include: per capita GDP, number of healthcare technicians per 1000 people (reflecting regional health resources), population density (reflecting population characteristics), density of medical institutions (reflecting medical market competition), and urbanization rate (reflecting counties’ economy, administration, and culture) [50, 51]. Efficiency is hospital technical efficiency scores and came from the input-oriented BCC-CCR DEA model. In this model, the selection of input and output variables was guided by previous empirical studies [49, 52–55] and systematic clustering analysis, depending on the availability of data in the Chongqing Regional Health Information Platform. Four input indicators and four output indicators were selected. The statistical characteristics of the input-output data are shown in Table 1. Remaining measurement variables of various levels of the multilevel growth model are shown in Table 2.

**Table 1:** Statistical characteristics of input-output indicators of DEA model

Indicators	Min.	Max.	Mean	Std. Dev.
Input				
Physical area of the hospital	806	136,000	21,088.68	23,472.26
Actual number of open beds	15	1470	342.66	307.36
Total fixed assets	1264	635,559	56,597.5	84,562.53
Number of healthcare technicians	18	1403	343.18	295.22
Output				
Total income	2490	942,523	139,816.37	144,548.81
Number of hospital bed rotations	16.43	137.41	50.82	19.9
Number of outpatient and emergency visits	9372	847,318	212,314.14	172,899.31
Number of discharged patients	93	66,737	13,425.69	12,824.62

**Table 2:** Measurement variables and interpretations at all levels

Variable code	Variable name	Description and measure of the variable
y	Efficiency	Hospital technical efficiency score
Level-1	Intra-hospital changes	
t	Time	Years 2012–2016 expressed as 0–4, respectively
FP	Government financial assistance	Annual financial input (unit: 10000 yuan)
A2	Physical area of the hospital	Physical area of the hospital (unit: square meter)
A3	Actual number of open beds	Actual number of open beds per year (unit: bed)
A6	Number of healthcare technicians	Annual number of hospital healthcare technical personnel (unit: person)
A11	Total fixed assets	Total annual fixed assets (unit: 10000 yuan)
V1	Daily charge per bed (Cost of every bed-day in hospital: yuan/ bed-day)	Hospitalization income (including medical and pharmaceutical) / actual occupancy bed-days
V7	Medical costs per 100-yuan medical income	(Medical business cost + management cost) / (medical income * 100)
V8	Total assets turnover	(Medical income + other income) / total assets
V9	Ratio of revenue and expenditure	Balance of revenue and expenditure / (medical income + [basic income, financial assistance, and other income]) * 100%
V10	Asset-liability ratio	Total liabilities / total assets * 100%
Level-2	Inter-hospital differences	
GHP	Hospital grade	Numbers 0–4 represent unrated, middle second-class, upper second-class, middle first-class, and upper first-class hospitals, respectively
THP	Hospital category	Taking general hospitals as the reference category, the two virtual variables are transformed into maternal and child healthcare hospitals (FY) and traditional Chinese medicine hospitals (ZY)
Level-3	Inter-county differences	
GDP	Per capita gross domestic product (GDP)	GDP / county population; measures a region’s economic development and standard of living
D1	Number of healthcare technicians per 1000 people (person)	Total healthcare technician population / (county population * 1000); measures the level of human resource investment and equity of the distribution of medical and health services
DP	Population density	Total population / county area; used to measure county health service needs
HP	Density of medical institutions	Total number of medical institutions in the area / county area; measures county medical institutions’ competition
CZ	Urbanization rate	Urban population / permanent population; used to measure social and cultural development of a county

Source: Chongqing Health Information Center, Chongqing Health and Family Planning Statistical Yearbook, and Chongqing Statistical Yearbook

### Model development

The model is developed in four main steps. To verify the existence of hierarchy in the data, we first establish an empty Eq. 1 with uninterpreted variables:

1 $$ {\displaystyle \begin{array}{l} Level-1:{Y}_{tij}={\pi}_{0 ij}+{e}_{tij}\\ {}\begin{array}{l} Level-2:{\pi}_{0 ij}={\beta}_{00j}+{r}_{0 ij}\\ {} Level-3:{\beta}_{00j}={\gamma}_{000}+{u}_{00j}\end{array}\end{array}} $$

Here, *Y* is the efficiency score of hospitals, *t* = 0,1,2,3…*,*represents the year; *i* = 1,2,3… indicates the hospital; *j* = 1,2,3… indicates the county; *γ*_*000*_ represents the average of total efficiency, and *e*_*tij*,_ *r*_*0ij*_, and *u*_*00j*_ represent random variations of levels 1, 2, and 3, respectively. *π*_*0ij*_ represents the five-year average efficiency of the *i* hospitals in the *j* counties, *β*_*00j*_ represents the average efficiency of the *j* counties.

To test for a significant increase in efficiency and variance in levels 2 and 3 and to determine the rate of this growth change, we established an unconditional growth model (random coefficient model, Eqs. 2 and 3). Level 1 includes only *T* (time variables), and levels 2 and 3 did not include any explanatory variables; we set the slope of the time variables to be random. Models of linear growth (Eq. 2) and nonlinear growth (Eq. 3) were established to investigate the possible growth curve of efficiency.

2 $$ {\displaystyle \begin{array}{l}\mathrm{Level}\hbox{-} 1:\kern1em {Y}_{tij}={\pi}_{0ij}+{\pi}_{1ij}\left({T}_{tij}\right)+{e}_{tij}\\ {}\mathrm{Level}\hbox{-} 2:\kern1em {\pi}_{0ij}={\beta}_{00j}+{r}_{0ij}\\ {}\kern4.8em {\pi}_{1ij}={\beta}_{10j}+{r}_{1ij}\\ {}\mathrm{Level}\hbox{-} 3:\kern1em {\beta}_{00j}={\gamma}_{00 0}+{u}_{00j}\\ {}\kern4.8em {\beta}_{10j}={\gamma}_{10 0}+{u}_{10j}\end{array}} $$

Here, *r*_*0ij*_ is an interceptor residual in Level 2, *r*_*1ij*_ is a slope residue residual in Level 2; *u*_*00j*_ is an interceptor residual in Level 3, *u*_*10j*_ is a slope residue residual in Level 3. *β*_*10j*_ is the average linear growth rate of efficiency in the *j* hospitals, and *γ*_*100*_ is the overall average linear growth rate of efficiency. The remaining parameters have the same meaning as Eq. 1.

3 $$ {\displaystyle \begin{array}{l}\mathrm{Level}\hbox{-} 1:\kern1.12em {Y}_{tij}={\pi}_{0 ij}+{\pi}_{1 ij}\left({T}_{tij}\right)+{\pi}_{2 ij}\left({T^2}_{tij}\right)+{e}_{tij}\\ {}\mathrm{Level}\hbox{-} 2:\kern1.12em {\pi}_{0 ij}={\beta}_{00j}+{r}_{0 ij}\\ {}\kern5em {\pi}_{1 ij}={\beta}_{10j}+{r}_{1 ij}\\ {}\kern5em {\pi}_{2 ij}={\beta}_{20j}+{r}_{2 ij}\\ {}\mathrm{Level}\hbox{-} 3:\kern1em {\beta}_{00j}={\gamma}_{000}+{u}_{00j}\\ {}\kern4.8em {\beta}_{10j}={\gamma}_{100}+{u}_{10j}\\ {}\kern4.8em {\beta}_{20j}={\gamma}_{200}+{u}_{20j}\end{array}} $$

Here, *β*_*20j*_ is the average nonlinear growth rate of efficiency in *j* hospital, *γ*_*200*_ is the average nonlinear growth rate of efficiency, and *u*_*20j*_ is the slope residue residual in Level-3. The remaining parameters have the same meaning as Eq. 2.

Based on Eq. 2 and Eq. 3, we include the explanatory variables at level 3 (Eq. 4) and level 2 (Eq. 5), and level 1 remained unchanged. To prevent multicollinearity between the interaction and original variable, explanatory variables at level 3 were grand-mean center processed. All variables at level 2 were categorical or hierarchical variables without centralization. Based on Eq. 5, the explanatory variables at level 1 were treated as group-mean centered and included to create Eq. 6. In Eqs. 4, 5 and 6, *γ*_*00p*_(*p* = 1,2,3 ... 5) is the slope of the influence of the level 3 interpretation variable on the initial efficiency score; *γ*_*10p*_(*p* = 1,2, 3 ...5) is the slope of the influence of the level 3 interpretation variables on the growth rate of efficiency; *β*_*0pj*_(*p* = 1,2,3,4) is the slope of the level 2 interpretation variable on the initial efficiency score; and *β*_*1pj*_(*p* = 1,2,3,4) is the slope of the level 2 interpretation variable on the efficiency growth rate. The remaining parameters have the same meaning as previous models.

4 $$ {\displaystyle \begin{array}{l}\mathrm{Level}\hbox{-} 1:\kern1em {Y}_{tij}={\pi}_{0 ij}+{\pi}_{1 ij}\left({T}_{tij}\right)+{e}_{tij}\\ {}\mathrm{Level}\hbox{-} 2:\kern1em {\pi}_{0 ij}={\beta}_{00j}+{r}_{0 ij}\\ {}{\pi}_{1 ij}={\beta}_{10j}+{r}_{1 ij}\\ {}\mathrm{Level}\hbox{-} 3:\kern.8em {\beta}_{00j}={\gamma}_{000}+{\gamma}_{001}(GDP)+{\gamma}_{002}\left(D 1\right)+{\gamma}_{003}(DP)+{\gamma}_{004}(HP)+{\gamma}_{005}(CZ)+{u}_{00j}\\ {}\kern.4em {\beta}_{10j}={\gamma}_{100}+{\gamma}_{101}(GDP)+{\gamma}_{102}\left(D 1\right)+{\gamma}_{103}(DP)+{\gamma}_{104}(HP)+{\gamma}_{105}(CZ)+{u}_{10j}\end{array}} $$

5 $$ {\displaystyle \begin{array}{l}\mathrm{Level}\hbox{-} 1:\kern1em {Y}_{tij}={\pi}_{0 ij}+{\pi}_{1 ij}\left({T}_{tij}\right)+{e}_{tij}\\ {}\begin{array}{l}\mathrm{Level}\hbox{-} 2:\kern1em {\pi}_{0 ij}={\beta}_{00j}+{\beta}_{01j}(GHP)+{\beta}_{02j}(IHP)+{\beta}_{03j}(ZY)+{\beta}_{04j}(FY)+{r}_{0 ij}\\ {}{\pi}_{1 ij}={\beta}_{10j}+{\beta}_{11j}(GHP)+{\beta}_{12j}(IHP)+{\beta}_{13j}(ZY)+{\beta}_{14j}(FY)+{r}_{1 ij}\\ {}\mathrm{Level}\hbox{-} 3:\kern1em {\beta}_{00j}={\gamma}_{000}+{\gamma}_{001}(GDP)+{\gamma}_{002}\left(D 1\right)+{\gamma}_{003}(DP)+{\gamma}_{004}(HP)+{\gamma}_{005}(CZ)+{u}_{00j}\\ {}{\beta}_{10j}={\gamma}_{100}+{\gamma}_{101}(GDP)+{\gamma}_{102}\left(D 1\right)+{\gamma}_{103}(DP)+{\gamma}_{104}(HP)+{\gamma}_{105}(CZ)+{u}_{10j}\end{array}\end{array}} $$

6 $$ {\displaystyle \begin{array}{l}\mathrm{Level}\hbox{-} 1:\kern1em {Y}_{tij}={\pi}_{0 ij}+{\pi}_{1 ij}\left({T}_{tij}\right)+{\pi}_{2 ij}\left({FP}_{tij}\right)+{\pi}_{3 ij}\left(A{2}_{tij}\right)+{\pi}_{4 ij}\left(A{3}_{tij}\right)+{\pi}_{5 ij}\left(A{6}_{tij}\right)\\ {}+{\pi}_{6 ij}\left(A{11}_{tij}\right)+{\pi}_{7 ij}\left(V{1}_{tij}\right)+{\pi}_{8 ij}\left(V{7}_{tij}\right)+{\pi}_{9 ij}\left(V{8}_{tij}\right)+{\pi}_{10 ij}\left(V{9}_{tij}\right)\\ {}+{\pi}_{11 ij}\left(V{10}_{tij}\right)+{e}_{tij}\\ {}\begin{array}{l}\mathrm{Level}\hbox{-} 2:\kern1em {\pi}_{0 ij}={\beta}_{00j}+{\beta}_{01j}(GHP)+{\beta}_{02j}(IHP)+{\beta}_{03j}(ZY)+{\beta}_{04j}(FY)+{r}_{0 ij}\\ {}\begin{array}{l}{\pi}_{1 ij}={\beta}_{10j}+{\beta}_{11j}(GHP)+{\beta}_{12j}(IHP)+{\beta}_{13j}(ZY)+{\beta}_{14j}(FY)+{r}_{1 ij}\\ {}\begin{array}{l}\mathrm{Level}\hbox{-} 3:\kern1.5em {\beta}_{00j}={\gamma}_{000}+{\gamma}_{001}(GDP)+{\gamma}_{002}\left(D 1\right)+{\gamma}_{003}(DP)+{\gamma}_{004}(HP)+{\gamma}_{005}(CZ)+{u}_{00j}\\ {}{\beta}_{10j}={\gamma}_{100}+{\gamma}_{101}(GDP)+{\gamma}_{102}\left(D 1\right)+{\gamma}_{103}(DP)+{\gamma}_{104}(HP)+{\gamma}_{105}(CZ)+{u}_{10j}\end{array}\end{array}\end{array}\end{array}} $$

To test the interaction effect between a single variable at level 1 and the time, the slope of each explanatory variable at level 1 is set separately as the cross-layer random slope. The results show that the random slope of all these variables is not significant at levels 2 and 3, and so we fix all their slopes. This study assumes that variables *FP*, *A2*, *A3*, *A6*, and *A11* affect the growth rate; that is, the relationship between time and efficiency. The interaction between these explanatory variables and time are included in the respective models, thus resulting in Eq. 7 (the full model).

7 $$ {\displaystyle \begin{array}{l}\mathrm{Level}\hbox{-} 1:\kern1em {Y}_{tij}={\pi}_{0 ij}+{\pi}_{1 ij}\left({T}_{tij}\right)+{\pi}_{2 ij}\left({FP}_{tij}\right)+{\pi}_{3 ij}\left(A{2}_{tij}\right)+{\pi}_{4 ij}\left(A{3}_{tij}\right)+{\pi}_{5 ij}\left(A{6}_{tij}\right)\\ {}+{\pi}_{6 ij}\left(A{11}_{tij}\right)+{\pi}_{7 ij}\left(V{1}_{tij}\right)+{\pi}_{8 ij}\left(V{7}_{tij}\right)+{\pi}_{9 ij}\left(V{8}_{tij}\right)+{\pi}_{10 ij}\left(V{9}_{tij}\right)\\ {}+{\pi}_{11 ij}\left(V{10}_{tij}\right)+{\pi}_{12 ij}\left({T}_{tij}\times A{2}_{tij}\right)+{\pi}_{13 ij}\left({T}_{tij}\times A{3}_{tij}\right) +_{14 ij}\left({T}_{tij}\times A{6}_{tij}\right)\\ {}+{\pi}_{15 ij}\left({T}_{tij}\times A{11}_{tij}\right)+{e}_{tij}\\ {}\begin{array}{l}\mathrm{Level}\hbox{-} 2:\kern1em {\pi}_{0 ij}={\beta}_{00j}+{\beta}_{01j}(GHP)+{\beta}_{02j}(IHP)+{\beta}_{03j}(ZY)+{\beta}_{04j}(FY)+{r}_{0 ij}\\ {}{\pi}_{1 ij}={\beta}_{10j}+{\beta}_{11j}(GHP)+{\beta}_{12j}(IHP)+{\beta}_{13j}(ZY)+{\beta}_{14j}(FY)+{r}_{1 ij}\\ {}\begin{array}{l}{\pi}_{pij}={\beta}_{p0j;}\\ {}\mathrm{Level}\hbox{-} 3:\kern1em {\beta}_{00j}={\gamma}_{000}+{\gamma}_{001}(GDP)+{\gamma}_{002}\left(D 1\right)+{\gamma}_{003}(DP)+{\gamma}_{004}(HP)+{\gamma}_{005}(CZ)+{u}_{00j}\\ {}{\beta}_{10j}={\gamma}_{100}+{\gamma}_{101}(GDP)+{\gamma}_{102}\left(D 1\right)+{\gamma}_{103}(DP)+{\gamma}_{104}(HP)+{\gamma}_{105}(CZ)+{u}_{10j}\\ {}{\beta}_{p0j}={\gamma}_{p00;}\end{array}\end{array}\end{array}} $$

Among them, *π*_*12ij*_ *、π*_*13ij*_ *、π*_*14ij*_ *、π*_*15i*j_ are the slope of the interaction items of *A2, A3, A6,* and *A11* with the time variable *T* on the initial efficiency. Other parameters have the same meaning as the preceding model.

## Results

### Descriptive results

Descriptive statistics were depicted for the hospitals’ efficiency scores and other continuous variables across the five measurement points (Tables 3 and 4). As seen in Table 3, the mean efficiency score was 0.79 in 2012 and 0.85 in 2016, exhibiting a trend that was growing at an increasing rate in Chongqing. Due to lack of the national average efficiency scores, we did literature review and found the score in 2012 was lower than those scores in other provinces [56–59], the changes over time indicate a diversifying trend of efficiency over the years.

**Table 3:** Descriptive statistics of continuous variables

Variable name	Years	Min.	Max.	Mean	Std. Dev.
Efficiency score	2012–2016	0.42	1.00	0.83	0.16
	2012	0.50	1.00	0.79	0.18
	2013	0.47	1.00	0.83	0.16
	2014	0.51	1.00	0.81	0.16
	2015	0.55	1.00	0.86	0.13
	2016	0.42	1.00	0.85	0.15
Government financial assistance	2012–2016	0.00	0.82	0.19	0.17
Physical area of the hospital		806.00	136,000.00	21,088.68	23,472.26
Actual number of open beds		15.00	1470.00	342.66	307.36
Number of healthcare technicians		18.00	1403.00	343.18	295.22
Total fixed assets		1264.00	635,559.00	56,597.50	84,562.53
Daily charge per bed (Cost of every bed-day in hospital: yuan/bed-day)		0.30	2.89	0.83	0.28
Medical costs per 100-yuan medical income		0.0009	0.0247	0.0106	0.0022
Total assets turnover		0.09	6.06	1.20	0.70
Ratio of revenue and expenditure		−3.686	98.018	22.45	16.37
Asset-liability ratio		0.00	102.03	44.95	25.21
Per capita gross domestic product (GDP)		12,969.47	91,552.13	37,720.33	19,792.18
Number of healthcare technicians per 1000 people (person)		1.70	17.70	4.42	2.73
Population density		0.01	0.40	0.07	0.10
Density of medical institutions		0.06	1.92	0.35	0.41
Urbanization rate		27.120	95.70	49.27	20.35

Note: The efficiency score is technical efficiency derived from the result of the DEA model. DEAP 2.1 software was used

**Table 4:** Level 2 descriptive statistics

GHP			THP		
Groups	N	Percentage (%)	Groups	N	Percentage (%)
Upper first-class hospital	30	8.3	General hospitals	120	33.3
Upper second-class hospital	230	63.9	Traditional Chinese medicine hospitals	120	33.3
Middle second-class hospital	65	18.1	Maternal and child healthcare hospitals	120	33.3
No grade	35	9.7			
Total	360	100.0		360	100.0

Note: *GHP* hospital grade, *THP* hospital category

### Analytic results

The results of Eq. 1 (Table 5) showed that the intraclass correlation coefficient (ICC) is 48.2% (ICC > 16%), indicating that 48.2% of total variance in average efficiency existed at level 2. For level 3, 6% < ICC < 16% (ICC = 8.1%), which indicated that 8.1% of the total variance in average efficiency exists at level 3 [60]. Based on this, the preliminary judgment was that there are statistically significant variance at both levels 2 and 3 [61]. Because the multilevel growth model considers both the initial efficiency and the slope, we must combine the significant test results of the increasing slope of *Y* in level 3 to judge the suitability of the three-level model.

**Table 5:** Results of all models

Level	Parameters and Variables	Model 1	Model 2	Model 3	Model 4	Model 5	Model 6	Model 7
Fixed Effect								
Level 1	Initial efficiency (π_0ij)_							
	Intercept (γ000)	0.829^***^ (0.017)	0.798^***^ (0.021)	0.792^***^ (0.022)	0.798^***^ (0.019)	0.843^***^ (0.068)	0.695^***^ (0.092)	0.823^***^ (0.061)
	Linear growth rate (T, π_1_ij)							
	Intercept (*β*_*100*_)		0.016** (0.005)	0.028* (0.011)				
	Nonlinear growth rate (*T2*, *π*_*2ij*_)			−0.003 (0.003)				
	Intercept (*β*_*200*_)							
	FP (*π*_*2ij*_)						0.337^**^ (0.103)	0.324^**^ (0.099)
	A2 (*π*_*3ij*_)						0.000^***^ (0.000)	0.000 (0.000)
	A3 (*π*_*4ij*_)						0.000 (0.001)	0.000 (0.001)
	A6 (*π*_*5ij*_)						−0.001 (0.003)	−0.006^*^ (0.002)
	A11 (*π*_*6ij*_)						0.000 (0.000)	0.000 (0.000)
	V1 (*π*_*7ij*_)						0.119^**^ (0.044)	0.114^**^ (0.042)
	V7 (*π*_*8ij*_)						−12.491** (4.575)	−10.366* (4.555)
	V8 (*π*_*9ij*_)						0.038^**^ (0.014)	0.041^**^ (0.013)
	V9 (*π*_*10ij*_)						−0.001 (0.001)	−0.001 (0.001)
	V10 (*π*_*11ij*_)						0.000 (0.001)	0.000 (0.001)
	T × A2 (*π*_*12ij*_)							0.000 (0.000)
	T × A3 (*π*_*13ij*_)							0.000 (0.001)
	T × A6 (*π*_*14ij*_)							0.003^**^ (0.001)
	T × A11 (*π*_*15ij*_)							0.000 (0.000)
Level 2	GHP (*β*_*01j*_)					−0.025 (0.024)	0.027 (0.025)	−0.042 (0.024)
	IHP (*β*_*02j*_)					0.019 (0.049)	0.037 (0.048)	0.043 (0.046)
	ZY (*β*_*03j*_)					−0.079^*^ (0.038)	− 0.078^*^ (0.037)	− 0.063 (0.035)
	FY (*β*_*04j*_)					0.073 (0.056)	0.097 (0.055)	0.117^*^ (0.052)
Level 3	GDP (*γ*_*001*_)				0.000 (0.000)	0.000 (0.000)	0.003 (0.003)	0.003 (0.003)
	D1 (*γ*_*002*_)				0.003 (0.021)	0.006 (0.024)	0.001 (0.025)	0.002 (0.026)
	DP (*γ*_*003*_)				1.105 (0.699)	0.753 (0.842)	1.216 (0.782)	1.224 (0.779)
	HP (*γ*_*004*_)				−0.103 (0.141)	−0.050 (0.149)	−0.092 (0.129)	− 0.105 (0.133)
	CZ (*γ*_*005*_)				−0.005 (0.003)	−0.004 (0.003)	− 0.005 (0.003)	−0.005 (0.003)
	Growth rate **(*T*,** ***π***_***0ij***_**)**							
	Intercept (γ_100_)		0.016^**^ (0.005)	0.028^*^ (0.011)	0.016^**^ (0.005)	0.020 (0.190)	0.023 (0.026)	0.025^*^ (0.013)
Level 2	GHP (*β*_*11j*_)					−0.001 (0.005)	0.001 (0.004)	0.004 (0.004)
	IHP (*β*_*11j*_)					0.003 (0.014)	−0.006 (0.013)	−0.009 (0.013)
	ZY (*β*_*11j*_)					0.013 (0.011)	0.011 (0.011)	0.008 (0.008)
	FY (*β*_*11j*_)					−0.019 (0.015)	−0.032 (0.018)	− 0.036^**^ (0.014)
Level 3	GDP (*γ*_*101*_)				0.000 (0.000)	0.000 (0.000)	−0.001 (0.001)	−0.001 (0.001)
	D1 (*γ*_*102*_)				0.007^*^ (0.003)	0.007^*^ (0.003)	0.009^*^ (0.004)	0.009^*^ (0.004)
	DP (*γ*_*103*_)				−0.075 (0.157)	− 0.095 (0.187)	−0.332^*^ (0.169)	− 0.310 (0.175)
	HP (*γ*_*104*_)				−0.016 (0.031)	−0.013 (0.034)	0.009 (0.029)	0.007 (0.029)
	CZ (*γ*_*105*_)				0.000 (0.001)	0.000 (0.001)	0.001 (0.001)	0.001 (0.001)
Variance Components								
*σ*^*2*^	Initial efficiency	0.011^***^ (0.001)	0.008^***^ (0.001)	0.007^***^ (0.000)	0.008^***^ (0.001)	0.008^***^ (0.001)	0.007^***^ (0.001)	0.006^***^ (0.001)
*σ*^*2*^_*r0*_	Initial efficiency	0.012^***^ (0.002)	0.021^***^ (0.004)	0.020^***^ (0.000)	0.021^***^ (0.004)	0.014^***^ (0.003)	0.013^***^ (0.003)	0.012^***^ (0.003)
	Growth rate		0.001^**^ (0.000)	0.004 (0.000)	0.001^**^ (0.000)	0.001 (0.000)	0.001^*^ (0.000)	0.000^*^ (0.000)
*σ*^*2*^_*u0*_	Initial efficiency	0.002 (0.002)	0.002 (0.003)	0.003 (0.003)	0.001 (0.004)	0.002 (0.003)	0.002 (0.002)	0.003 (0.003)
	Growth rate		0.000** (0.000)	0.000 (0.000)	0.000 (0.000)	0.000 (0.000)	0.000 (0.000)	0.000 (0.000)
Covariance								
Level 2	Initial efficiency and growth rate		−0.003^**^ (0.001)	−0.002 (0.002)	−0.003^**^ (0.001)	−0.002 (0.001)	−0.001 (0.001)	−0.001 (0.001)
Level 3			0.000 (0.000)	−0.001 (0.001)	0.000 (0.001)	0.000 (0.001)	0.000 (0.000)	0.000 (0.001)
Model fit	Np	4	9	16	19	27	37	41
	LL	228.239	247.529	250.324	251.646	260.718	292.292	302.857
	AIC	− 448.490	− 477.059	− 468.649	−465.291	−467.436	− 510.584	−523.713
	BIC	− 432.946	− 442.084	− 406.471	− 392.455	− 362.511	−366.798	− 364.383
ICC	Level 2	0.482						
	Level 3	0.081						

Note: * *p* < 0.05, ** *p* < 0.01, and *** *p* < 0.001. *Np* the model estimation parameter, *LL* logarithmic likelihood ratio, *AIC* Akaike information criterion, *BIC* Bayesian information criterion, *ICC* intraclass correlation coefficient

The results of Eqs. 2 and 3 (Table 5) showed that the average growth rate of the linear growth model was significant (*β* = 0.016). There was a significant, negative correlation between the linear growth rate and initial efficiency (*r* = − 0.003), indicating that the lower the initial efficiency score, the higher the growth rate. However, the primary (linear) growth rate of the nonlinear growth model was significant (*β* = 0.028) and the secondary (nonlinear) growth rate was not significant (*β* = − 0.003), suggesting that linear growth is maintained. In addition, the growth variance of the linear growth model was significant at levels 2 and 3, but variance of the primary and secondary growth rates in the nonlinear growth model were not significant. This suggests that the growth rate of only the linear growth model differs between hospitals and counties. In addition, judging from the model-fitting, the Akaike and Bayesian information criteria of the linear growth model were lesser than those of the nonlinear growth model, indicating that the linear growth model fitted better. Judging from the above, county public hospital efficiency exhibits linear growth, and this growth was different for different hospitals and counties. Thus, the multilevel linear growth model for the “time-hospital-county” was suitable in this context.

**Fig. 2:**
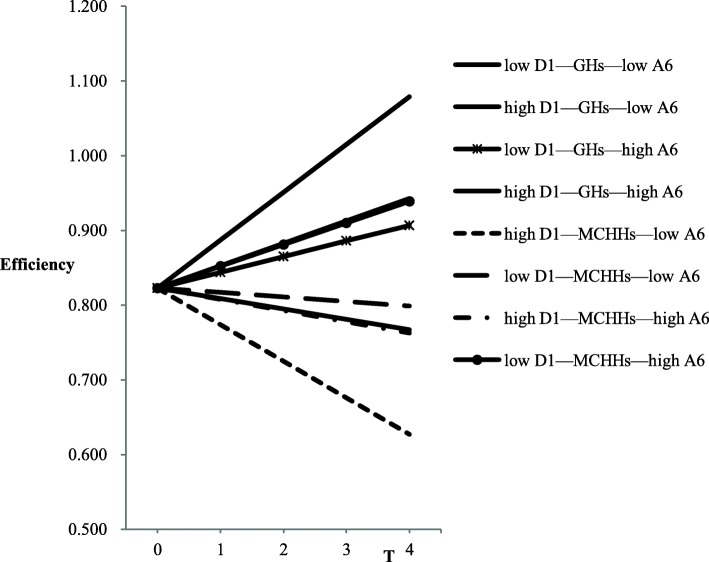
Three-level interaction effects. A6 = number of healthcare technicians; D1 = number of healthcare technicians per 1000 people; GH = general hospital; MCHH = maternal and child healthcare hospital. The variables take “mean−1 standard deviation” as the low level and “mean+1 standard deviation” as the high level

Adding variables in turn from level 3 to level 1 resulted in Eqs. 4, 5 and 6,^4^ and model 7. According to the results of the full model (Eq. 7) in Table 5, *FP* (*β* = 0.324), *V1* (*β* = 0.114), and *V8* (*β* = 0.041) had significant, positive effects on the initial efficiency, while *A6* (*β* = − 0.006) and *V7* (*β* = − 10.366) had significant, negative effects. In level 2, the influence of the variable *GHP* on the initial efficiency was not significant, and the initial efficiency of MCHHs was significantly higher than that of GHs (*β* = 0.11). However, there was no significant difference between the initial efficiency score of TCMHs and GHs, and no variables at level 3 had significant impacts on initial efficiency. In terms of growth rate, the efficiency growth rate of MCHHs at level 2 was significantly lower than that of GHs (*β* = − 0.036). The *GHP* had no significant impacts on the growth rate, and there was no significant difference in the efficiency growth rate between TCMHs and GHs. The variable *D1* in level 3 had a significant, positive effect on the growth rate (*β* = 0.009). However, the impact of *GDP*, *DP*, *HP*, and *CZ* on the growth rate was not significant. Results of the simultaneous interaction were shown in Table 5 and depicted a significant increase in hospital efficiency (*β* = 0.025); that is, an average annual increase of 0.025. At level 1, only the interaction of *A6* and *T* were significant (*β* = 0.003). In addition, *THP* (*FY*) and *D1* also had significant impacts on the growth rate. To better understand the results of the final model (Eq. 7), we showed the results graphically in Fig. 2. The graphs displayed the model-based trajectories for *A6, THP,* and *D1*, further subdivided by high and low levels, which were defined as one standard deviation (SD) above and below the mean, respectively. According to the results of Table 6, when *D1* and *A6* are high, the efficiency of the GHs increases significantly (the slope was 0.064, with an average annual increase of 0.064). For a high *D1* and low *A6*, the efficiency of the MCHHs decreased significantly (the slope was − 0.049, with an average annual decrease of 0.049).

**Table 6:** Simple slope of the model adjusted simultaneously

Group	Simple slope equation	Significance of slope (p)
low D1—GH_S_—low A6	y = −0.014 T + 0.823	0.482
high D1—GH_S_—low A6	y = 0.030 T + 0.823	0.091
low D1—GH_S_—high A6	y = 0.021 T + 0.823	0.184
high D1—GH_S_—high A6	y = 0.064 T + 0.823	0.000***
high D1—MCHH_S_—low A6	y = − 0.049 T + 0.823	0.002**
low D1—MCHH_S_—low A6	y = − 0.006 T + 0.823	0.694
high D1—MCHH_S_—high A6	y = − 0.015 T + 0.823	0.294
low D1—MCHH_S_—high A6	y = 0.029 T + 0.823	0.097

Note:^**^ *p* < 0.01, and ^***^ *p* < 0.001. Number of healthcare technicians (A6) and number of healthcare technicians per 1000 people (D1) take “mean − 1 standard deviation” as the low level and “mean + 1 standard deviation” as the high level. *GHs* general hospitals, *MCHHs* maternal and child healthcare hospitals

## Discussion

Effective health promotion measures are a crucial part of bringing the differences of efficiency in the initial efficiency and linear growth between hospitals and counties in Chongqing into parity. The results presented in section 4 provide timely implications.

### Inter-County level implications

Previous studies suggest that greater competition is positively associated with efficiency [13]. However, the present study finds that *HP* does not affect the initial score and growth rate of hospital efficiency. Possible explanation of this result is that a county’s medical market competition cannot function well as a market that allocates resources due to the existence of monopoly, externality, etc. Existing health resources of hospital are still far from meeting the service requirements. Further analysis is needed to confirm this explanation. Moreover, *D1* has a significant, positive impact on the growth rate of efficiency. Combined with the theory of economies of scale, it should take into account the service radius, population density, amount of services that hospitals can actually carry, and competition between county medical institutions in expanding the resources and scale to avoid blind development. Therefore, maintaining the stability of the current number and reasonable increase in the number of new county health technicians is significant to the next reform according to model results.

Geographical locations are also significant determinants of efficiency [19], although this study illustrates that the impacts of *GDP* and *CZ* on the growth rate are not significant. This is in contrast with the traditional relationship between hospital service efficiency and the level of economic development seen in the past, and it provides another perspective for future researchers other than the level of counties’ economic development.

### Inter-hospital level implications

This study shows that *GHP* has no effect on the initial efficiency and growth rate of hospital efficiency. The initial efficiency score for MCHHs is higher than that for GHs, and there is no significant difference in the initial efficiency and growth rate between TCMHs and GHs. However, the growth rate of MCHHs is significantly lower than that of GHs. This means that, in 2012, the efficiency of MCHHs was relatively high in Chongqing, but efficiency growth between 2012 and 2016 was significantly lower for GHs and TCMHs. The reason may be that, since the new medical reform, the state has reformed GHs and TCMHs more vigorously to ensure they are fully functional and effective. Meanwhile, the construction of a traditional medicine services system has been incorporated into regional health plans. As the main entities of this system, TCMHs have increased investment in infrastructure and personnel, strengthened discipline development, vigorously promoted appropriate technologies, improved their ability to innovate in science and technology, and improved their overall efficiency. Further reform should focus on MCHHs for increasing investment and implementing government compensation.

### Intra-hospital level implications

*FP* has a positive impact on the initial efficiency of public hospitals at level 1, which is in line with a previous study [62]. One reasonable explanation is that the General Office of the State Council issued the “*Guidance on the Comprehensive Reform of Urban Public Hospitals*” on May 17, 2015 (National Office, 2015, No. 38) and proposed that the compensation channel for public hospitals should be changed from original service charges, income from the sale of drugs, and government subsidies to service charges and government subsidies. As an important source of compensation, government subsidies have a positive impact on the operation of public hospitals.

In a review of the literature on medical personnel, Tsekouras et al. [19] find that medical personnel are crucial for the improvement of public hospitals’ productive efficiency. Results of the present study show that A6 has a significant, negative impact on the initial efficiency. This is consistent with the information revealed by the iutput-oriented DEA model, which hints that the number of health technicians in the hospital must be reduced to be more efficient in a given output, and also match the principles of basic microeconomics.

Moreover, *V7* has a significant, negative effect on the initial efficiency. The “*Guidance of the General Office of the State Council on the Pilot Reform of Urban Public Hospitals*” (National Office, 2015, No. 38) notes that, by 2017, the consumption of health materials in *V7* of public hospitals in pilot cities will be reduced to less than 20 yuan. This indicator is used as a key factor in the cost control of hospitals. The control of the consumption ratio of 100-yuan medical income has promoted the rationalization and adjustment of the medical income structure, optimized the level of hospitals’ organizational efficiency, effectively promoted the structural adjustment of medical income, and improved hospital efficiency [63].

### Implications of the interaction of variables

An important implication of the results is that *A2, A3, A6,* and *A11* separately affect the relationship between *T* and efficiency, which can significantly affect growth rates. When these four interaction items are included simultaneously, there is a significant increase in efficiency, with an average annual increase of 0.025. However, only *D1, THP,* and *A6* remain significant, and interactions of other terms should be explored in future research with a larger sample. The stability and increase of *D1* in a county are factors that improve the efficiency of county-level hospitals in Chongqing.

Finally, it should be noted that the situation of county-level public hospitals in different counties of Chongqing is more complex, and the limited factors in this model cannot fully explain the growth trajectory of hospital efficiency or the differences. The results of the analysis show that after considering the relevant factors of hospital and county characteristics, some indicators still exhibit significant differences between hospitals and counties. Some hospital-specific indicators, such as *FP, V1,* and *V8*, also vary between hospitals or counties. The difference is mainly reflected in the initial efficiency rather than the growth rate. That is, these differences in indicators can be explained by factors at the hospital level. In this study, the variation of hospital efficiency is decomposed into the difference among Intra-hospital, Inter-hospitals, and Inter-counties so that the random error of individuals is purer, and the obtained parameters are more accurate. The reasons for the difference in hospital efficiency can be more rationally analyzed and explained. As studies on hospital efficiency have infrequently used multilevel growth models, the results of related studies should be interpreted and treated with significant caution. The following are noteworthy limitations of this study. First, the sample size for this study is relatively insufficient, and some meaningful results have not been observed. Although, results of the study have good robustness to the change in the efficiency trend, because five rounds of panel data were selected [44]. Second, this study is limited by the variables available in the Chongqing Information Platform. Other variables not incorporated in the model include the health policy system, health service needs, responsiveness, and health status of the population, which can be collected through interviews, questionnaires, etc. The study’s implications are relatively limited, which limits the pertinence and reliability of the research conclusions. Third, reform itself is really a non-ignorable factor for hospital efficiency change over time. Since it is not the focus of this paper, it is not taken into account in this research design. Discussing the impact of reform on efficiency will be a meaningful direction for future studies.

Despite these limitations, this study is a useful preliminary study, based on the existing information system platform, to explore factors over time and at different levels in Chinese county-level hospitals during the period of new healthcare reform.

## Conclusion

This study describes and demonstrates the importance of examining both hospital and county characteristics related to efficiency over time. With the application of multilevel analysis techniques, the method of hospital efficiency evaluation has undergone a fundamental change. A three-level growth model describes relationships between time, hospital characteristics, and county characteristics. In this study, the variation of hospital efficiency is decomposed into different levels, which increases the purity of the random error of individuals, and the parameter estimation is more accurate. The reasons for the difference in hospital efficiency can be analyzed and explained more accurately. The results of this study illustrate that per capita GDP has no significant effect on efficiency, but government financial assistance to hospitals has a significant, positive effect. This is because counties with a developed economy can guarantee financial subsidies to hospitals. There is a possibility that a county’s economy indirectly affects hospital efficiency through fiscal aid. Therefore, there is a need within this field for further studies that use a large sample. Combined with the above results, the stability and reasonable increase in the number of healthcare technicians in a county are the key factors that improve the efficiency of county-level hospitals in Chongqing. Further reform should focus on MCHHs for increasing investment and implementing government compensation.

## Data Availability

All data generated or analyzed in this study are included in this published study and the additional tables. The used statistical data during this research are available on the official website of the Chongqing Health Statistics, http://tj.cqshic.com/category.aspx?id=5c4e162b-e84b-4964-9b43-83f2a38d58ed The datasets and materials during the current study are available from the corresponding author on reasonable request.

## Abbreviations

DEA: Data envelopment analysis
GDP: Gross Domestic Product
GHs: General hospitals
ICC: Intraclass correlation coefficient
MCHHs: Maternal and child healthcare hospitals
SD: Standard deviation
TCMHs: Traditional Chinese medicine hospitals

## Footnotes

1 In this figure, horizontal arrows represent the role of a horizontal variable, and diagonal arrows represent the interaction between horizontal variables.

2 The minimum sample capacity of the DEA model is 2*N*M, where N, M represent the number of inputs and output indicators, respectively.

3 In China, the medical costs per 100-yuan medical income is measured as follows: (medical business cost + administrative cost) / (medical income * 100) and is used to measure hospital cost management.

4 Models 4, 5, 6 were estimated, but their results were not discussed due to space limitations.
